# A nanocomplex that is both tumor cell-selective and cancer gene-specific for anaplastic large cell lymphoma

**DOI:** 10.1186/1477-3155-9-2

**Published:** 2011-01-31

**Authors:** Nianxi Zhao, Hitesh G Bagaria, Michael S Wong, Youli Zu

**Affiliations:** 1 Department of Pathology, the Methodist Hospital and the Methodist Hospital Research Institute, 6565 Fannin street, Houston, TX 77030, USA; 2 Departments of Chemical and Biomolecular Engineering and Chemistry, Rice University, 6100 Main Street, Houston, TX 77005, USA

## Abstract

**Background:**

Many *in vitro *studies have demonstrated that silencing of cancerous genes by siRNAs is a potential therapeutic approach for blocking tumor growth. However, siRNAs are not cell type-selective, cannot specifically target tumor cells, and therefore have limited *in vivo *application for siRNA-mediated gene therapy.

**Results:**

In this study, we tested a functional RNA nanocomplex which exclusively targets and affects human anaplastic large cell lymphoma (ALCL) by taking advantage of the abnormal expression of CD30, a unique surface biomarker, and the anaplastic lymphoma kinase (ALK) gene in lymphoma cells. The nanocomplexes were formulated by incorporating both ALK siRNA and a RNA-based CD30 aptamer probe onto nano-sized polyethyleneimine-citrate carriers. To minimize potential cytotoxicity, the individual components of the nanocomplexes were used at sub-cytotoxic concentrations. Dynamic light scattering showed that formed nanocomplexes were ~140 nm in diameter and remained stable for more than 24 hours in culture medium. Cell binding assays revealed that CD30 aptamer probes selectively targeted nanocomplexes to ALCL cells, and confocal fluorescence microscopy confirmed intracellular delivery of the nanocomplex. Cell transfection analysis showed that nanocomplexes silenced genes in an ALCL cell type-selective fashion. Moreover, exposure of ALCL cells to nanocomplexes carrying both ALK siRNAs and CD30 RNA aptamers specifically silenced ALK gene expression, leading to growth arrest and apoptosis.

**Conclusions:**

Taken together, our findings indicate that this functional RNA nanocomplex is both tumor cell type-selective and cancer gene-specific for ALCL cells.

## Background

The discovery of RNA interference (RNAi), the process by which specific mRNAs are targeted for degradation by complementary small interfering RNAs (siRNAs), has enabled the development of methods for the silencing of specific genes at the cellular level [1-3]. *In vitro *studies demonstrated that siRNA-mediated silencing of oncogenes induces growth arrest and death of tumor cells, indicating their potential therapeutic value [4-7]. Although siRNAs are gene specific, they are not cell/tissue-selective and therefore can not specifically target or accumulate in tumor tissues. Therefore, an efficient cell/tissue-specific delivery system is needed to make siRNA-mediated gene therapy a feasible approach. *In vivo *delivery of functional RNAs can be achieved using either viral carriers or non-viral cationic vectors. Although viral carriers achieve high transfection efficiencies, concerns about their safety, immunogenicity, and latent pathogenic effects have convinced researchers to focus on non-viral cationic carriers [8-11]. Among these cationic carriers, polyethyleneimine (PEI) has been widely studied due to its high cell transfection efficiency, strong buffering capacity, and ability to release functional nucleic acids from endosomes into the cytoplasm by inducing osmotic endosomal rupture [12-19]. However, PEI carriers alone are not cell/tissue-type specific, thus reaching tumor sites *in vivo *requires high treatment dosages of PEI, which may be toxic to normal tissues [20,21]. This cytotoxicity of PEI has thus far prevented its translation to the clinic [22]. While efforts to synthesize safer PEI analogues are underway, decreasing the required dosage of PEI could also reduce toxicity.

To gain cell specificity, the siRNA delivery system can be combined with a target-specific ligand molecule [23-26]. Although monoclonal antibodies have been widely used as cell-targeting ligands, mouse monoclonal antibodies are immunogenic *in vivo *and humanized monoclonal antibodies are very costly and only available for a limited number of ligands. Thus, scientists have searched for other ligand molecules that are easier to produce. Aptamers, short single-stranded oligonucleotides (30-50 bases) represent one such class of new small molecule ligands. In contrast to antibodies, aptamers are small oligonucleotides that exhibit no or minimal antigenicity/immunogenicity, so they are more suitable for *in vivo *use as diagnostic or therapeutic agents [27-29]. Recently, a RNA aptamer was developed that specifically binds to the CD30 protein in solution [30]. In addition, we have shown that this RNA aptamer selectively binds to intact CD30-expressing lymphoma cells with binding characteristics similar to a CD30-specific antibody [31].

Anaplastic lymphoma kinase (ALK)-positive anaplastic large cell lymphoma (ALCL) is an aggressive T-cell lymphoma [32-34]. ALCL cells exhibit an abnormal expression of the ALK oncogene and unique surface expression of CD30 [35-37]. The presence of these distinct molecular markers provides the rationale for development of a lymphoma cell-selective and tumor gene-specific therapeutic approach to treat ALCL. Previous studies demonstrated that siRNA-mediated knockdown of ALK gene expression promotes cell death of ALCL cells [38-40]. Based on these findings, we hypothesized that ALCL-selective delivery of a tumor gene-specific siRNA could be developed by assembling a functional RNA nanocomplex comprised of the CD30-specfic aptamer and an ALK-targeted siRNA within nano-sized PEI polymer carriers.

## Results

### Formulation of a nanocomplex containing both CD30 aptamer and ALK siRNA

**Figure 1:**
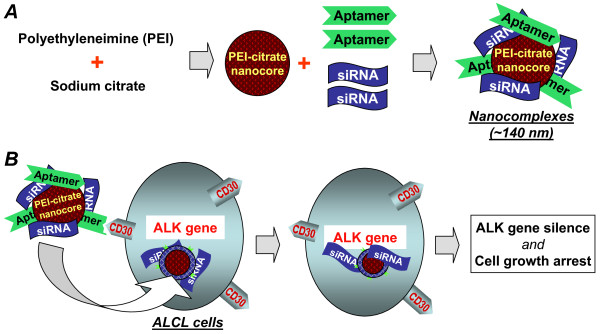
**Development of a tumor cell type-selective and cancer gene-specific nanocomplex for ALCL cells**. **A**, A nano-sized carrier core structure was initially formed *via *aggregation of polyethyleneimine (PEI) and crosslinking with sodium citrate (PEI-citrate nanocore). The synthetic RNA-based CD30 aptamers and ALK siRNA were then incorporated onto the PEI-citrate nanocore to form the nanocomplex. **B**, When the functional RNA nanocomplex is added to cultures, the aptamer component will selectively target CD30-positive ALCL cells. Aptamer-mediated cell binding will facilitate intracellular delivery of the nanocomplex. The siRNA component will subsequently silence the cellular ALK gene, resulting in the growth arrest of ALCL cells.

Briefly, the nanocomplexes were assembled by incorporating the synthetic siRNA and CD30 aptamer into the nano-sized carrier structure of PEI-citrate nanocores (Figure 1A). The rationale for our nanocomplex design is the CD30 aptamer provides selective binding of nanocomplexes to ALCL cells. The aptamer-mediated binding results in intracellular delivery of the ALK-targeted siRNA component exclusively into ALCL cells and subsequent silencing of the cellular ALK gene (Figure 1B).

**Figure 2:**
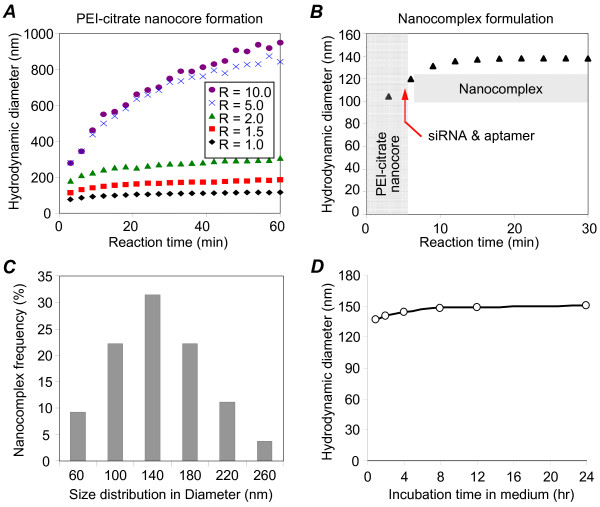
**Formulation of nanocomplex**. **A**, Dynamic light scattering (DLS) measurement of PEI-citrate nanocores, which were formed using different 'R' ratios of citrate to PEI (charge/charge). **B**, Assembly of the nanocomplexes by incorporation of PEI-citrate nanocores with synthetic ALK siRNA and CD30 aptamers. The arrow indicates the addition of siRNA and aptamer components into the reaction mix. The size of the nanocomplexes formed was measured over time by DLS. **C**, The frequency of the nanocomplexes with different sizes was calculated. **D**, Nanocomplexes were incubated in cell culture medium, and the sizes were measured over time by DLS.

First, the nanocore structure was formed by electrostatic crosslinking of positively-charged PEI with negatively-charged sodium citrate [41,42]. As shown in Figure 2A, the size of the PEI-citrate nanocores depended on the ratio ('R') of citrate to PEI (charge/charge) and the reaction time. For the present study a 'R' ratio of 1:1.5 with a reaction time of 5 minutes was chosen to obtain an ~120-nm PEI-citrate nanocore (Figure 2B). At the end of 5 minutes, the synthetic CD30 aptamers and ALK siRNAs were incorporated into the PEI-citrate nanocore carriers *via *non-covalent bonds to form a nanocomplex with a peak hydrodynamic diameter of ~140 nm (Figure 2B). However, the distribution of nanocomplex size ranged from 60 to 260 nm with approximately 80% of nanocomplexes being 100 to 180 nm in diameter (Figure 2C). The size of formed nanocomplexes was also confirmed by transmission electron microscopy (Additional File 1). Finally, the size of nanocomplexes remained stable in cell culture medium at room temperature for 24 hours (Figure 2D), demonstrating colloidal stability of the nanocomplex.

Zeta potential measurements show that the positive charge of PEI-citrate nanocore (+10 ± 0.6 mV) was reversed after incorporation of the siRNA and aptamer (-41 ± 0.9 mV). This was expected because of the negatively-charged nature of both the siRNA and aptamer that have complexed with the PEI-citrate nanocore. Further, the potential of the nanocomplex dropped to -25 ± 0.9 mV in the cell culture medium due to the high ionic strength.

**Figure 3:**
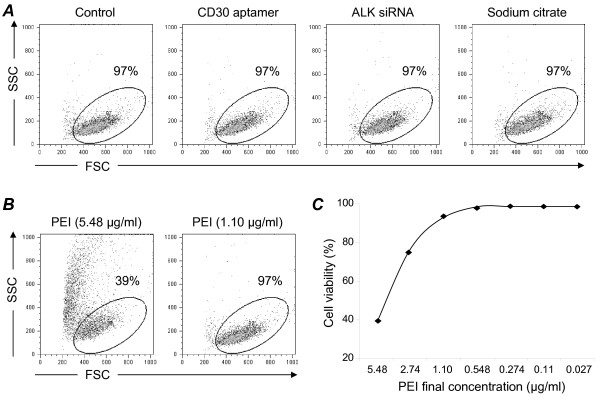
**Cytotoxicity assays of individual nanocomplex components**. **A**, Cultured Karpas 299 cells were treated for 48 hours with the individual components of the nanocomplex at their maximal concentrations: 100 nM CD30 aptamer, 100 nM ALK siRNA, and 4.2 μM sodium citrate, or vehicle only for the control group. Cell viability (%) was evaluated by flow cytometry using forward scatter (FSC) and side scatter (SSC) parameters as indicated. **B**, Karpas 299 cells were treated with PEI at concentrations of 5.48 and 1.10 μg/ml for 48 hours, and viable cells were quantified by flow cytometry, as above. **C**, Cell viability studies using serially diluted PEI ranging from 0.027 to 5.48 μg/ml.

To evaluate whether nanocomplexes, or their components, caused non-specific cytotoxicity, cultured Karpas 299 cells were treated for 48 hours with the individual nanocomplex components at their maximal concentrations. After treatment, cell viability was evaluated by flow cytometry. Treatment with 100 nM CD30 aptamer, 100 nM ALK siRNA, or 4.2 μM sodium citrate had no effect on cell viability (Figure 3A). Previous *in vitro *studies showed that high concentrations of PEI may be toxic to cells [20,21]. To determine a non-cytotoxic concentration of PEI, we treated Karpas 299 cells with serial dilutions of PEI for 48 hours and monitored cell viability by flow cytometry. As shown in Figure 3B, 5 μg/ml PEI was cytotoxic, significantly reducing cell viability. However, the observed cytotoxicity decreased as the PEI concentration decreased. Cytotoxicity was undetectable with treatment of ≤1.10 μg/ml PEI (Figure 3C). Thus, to rule out any PEI-mediated non-specific cellular effects, nanocomplexes made with a final PEI concentration of 0.274 μg/ml were used for further experiments. When used as a non-specific carrier for cell gene delivery, a final PEI concentration of 5-10 μg/ml was commonly used, a dose showed to be highly cytotoxic [15-21].

### CD30 aptamers mediate selective ALCL cell binding and intracellular delivery of nanocomplexes

**Figure 4:**
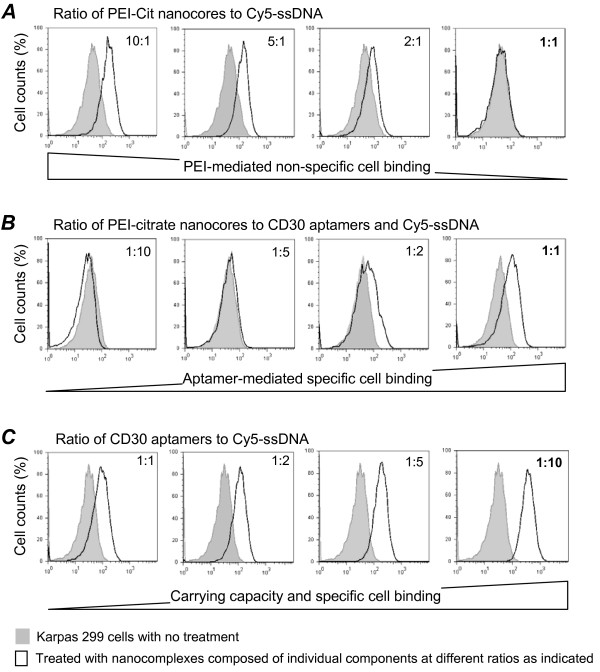
**Optimization of the specific cell binding and carrying capacity of the nanocomplexes**. **A**, Synthetic Cy5-conjugated ssDNA reporter molecules were incorporated into the PEI-citrate nanocores at different ratios (moles of nitrogen in PEI to moles of phosphate in ssDNA) as indicated. Reduction of the PEI-medicated non-specific cell binding was then monitored by flow cytometry. **B**, To gain specific cell binding capacity, the CD30 aptamer was incorporated into PEI-citrate nanocores along with the Cy5-ssDNA reporter to form a test nanocomplex. Different ratios of PEI-citrate nanocores to total oligonucleotides (moles of nitrogen in PEI/total moles of phosphate from both the aptamer and ssDNA) were tested as indicated, while the aptamer and Cy5-ssDNA were used at a fixed ratio of 1:1 (mol/mol). The CD30 aptamer-mediated specific binding to Karpas 299 cells was confirmed using flow cytometry. **C**, To optimize the maximal carrying capacity, the nanocomplex was formulated using a fixed ratio of PEI-citrate nanocores to total oligonucleotides (1:1 ratio as showed in **B**), but the ratios of the CD30 aptamer and Cy5-ssDNA reporter were altered as indicated (mol/mol). The carrying capacity of Cy5-ssDNA reporter by the nanocomplex with specific binding to Karpas 299 cells was quantified using flow cytometry.

First, Cy5-conjugated ssDNA corresponding to the sense sequence of the ALK siRNA was incorporated into nanocores at different ratios and used as a reporter for PEI-medicated non-specific cell binding. These reporter nanocomplexes were incubated with Karpas 299 cells for 30 minutes, and the resultant non-specific cell binding was quantified by flow cytometry. As shown in Figure 4A, PEI-mediated non-specific cell binding could be modulated by altering the ratio of incorporated ssDNA and was completely eliminated when the ratio of PEI to ssDNA (moles of nitrogen in PEI to moles of phosphate in ssDNA) was ≤ 1:1. Subsequently, to gain selective cell binding, the CD30 aptamer was incorporated into the PEI carrier along with the Cy5-ssDNA to form new test nanocomplexes. Different ratios of PEI carrier to total oligonucleotides were tested as indicated, but the CD30 aptamer and Cy5-ssDNA were used at a fixed ratio of 1:1 (mol/mol). As shown in Figure 4B, the highest specific binding to Karpas 299 cells was observed when a 1:1 ratio of PEI carrier to total amounts of aptamer and ssDNA (moles of nitrogen in PEI to total moles of total phosphate from both the aptamer and ssDNA) was used. Finally, to optimize the carrying capacity, the nanocomplexes were formulated using a fixed 1:1 ratio of PEI carrier to total oligonucleotides as described above, but the ratios of the CD30 aptamer and Cy5-ssDNA (mol/mol) were altered. A maximal carrying capacity of ssDNA by the nanocomplex was demonstrated when the CD30 aptamer and ssDNA were used at a ratio of 1:10 (Figure 4C).

**Figure 5:**
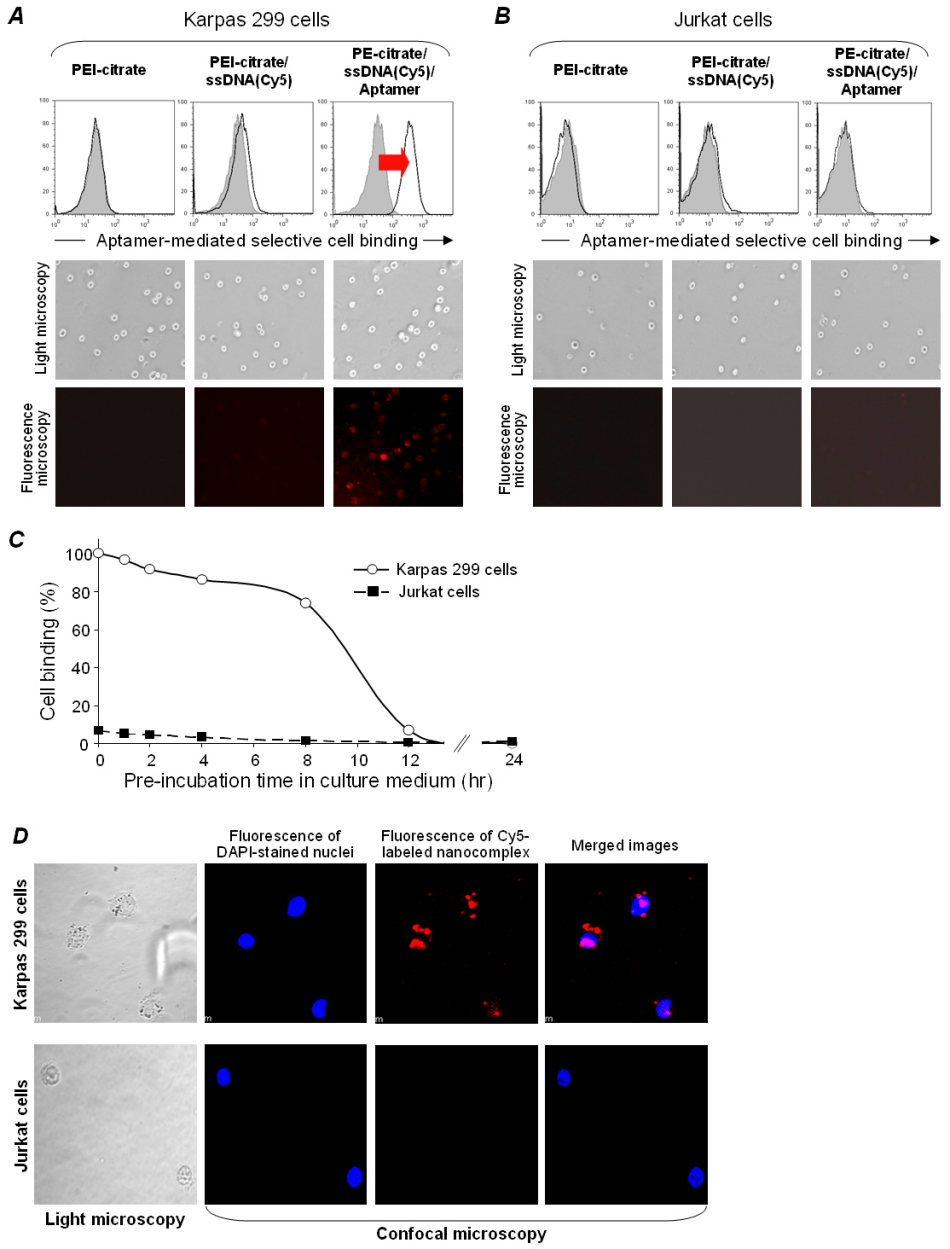
**CD30 aptamer-mediated selective cell binding and intracellular delivery of nanocomplexes**. **A**, Cultured Karpas 299 cells were treated with nanocomplexes containing the CD30 aptamer and Cy5-ssDNA reporter. Specific cell binding was detected by flow cytometry (top row), as well as fluorescence microscopy (bottom row) paired with light microscopy (middle row). To rule out non-specific cell binding, PEI-citrate nanocores alone or PEI-citrate nanocores containing the Cy5-ssDNA reporter (but no CD30 aptamer component) were used in control cultures. **B**, Cultured CD30-negative Jurkat cells were tested under the same treatment conditions. **C**, To assess functional biostability, the nanocomplex was pre-incubated in culture medium for up to 24 hours and changes in its cell binding capacity was kinetically monitored (%). CD30-negative Jurkat cells were used as a background binding control. **D**, To detect intracellular delivery, Karpas 299 cells (top row) and control Jurkat cells (bottom row) were treated with the nanocomplex containing both Cy5-ssDNA reporter and CD30 aptamer for 4 hours followed by quick nuclear staining with DAPI. As indicated, the treated cells were examined using light and confocal microscopy to visualize the DAPI-stained nuclei (blue) and the Cy5 reporter signal of the nanocomplex (red). Merged images of the DAPI-stained nuclei and Cy5 reporter signal indicate the intracellular localization of the nanocomplex.

For further confirmation, Karpas 299 cells and CD30-negative Jurkat cells were treated with PEI carrier alone, PEI carrier incorporated with the Cy5-ssDNA reporter (but no CD30 aptamer), or nanocomplexes carrying both the CD30 aptamer and the Cy5-ssDNA reporter. The resultant cell binding was monitored by flow cytometry (Figure 5). The presence of the CD30 aptamer, nanocomplexes selectively bound Karpas 299 cells, but not to Jurkat cells, which do not express the CD30 ligand (Figure 5A and 5B). In addition, aptamer-mediated CD30 selective binding of the nanocomplexes to Karpas 299 cells was also confirmed by fluorescent microscopy. Finally, the biostability of the nanocomplexes was evaluated by pre-incubating the nanocomplexes in culture medium and then adding Karpas 299 or Jurkat cells at different time points as indicated in Figure 5C. Specific cell binding of the nanocomplexes was then quantified by flow cytometry. The nanocomplex was functionally stable in culture medium and retained ~75% of its cell binding capacity after pre-incubation for 8 hours, but after 12 hours the binding capacity decreased to ~10% (Figure 5C).

To determine whether binding of the CD30 aptamer-mediated to the cell surface induced nanocomplex internalization, cells were incubated with the test nanocomplexes for 4 hours, the nuclei stained with DAPI, and examined by confocal microscopy. As shown in Figure 5D, intracellular delivery of the nanocomplexes was confirmed by observing the overlap of the Cy5-ssDNA reporter (red) with the DAPI-stained cell nuclei (blue). Control experiments using identically treated Jurkat cells showed no cellular binding or intracellular delivery of the nanocomplex.

### Nanocomplexes introduce functional siRNAs into ALCL cell

**Figure 6:**
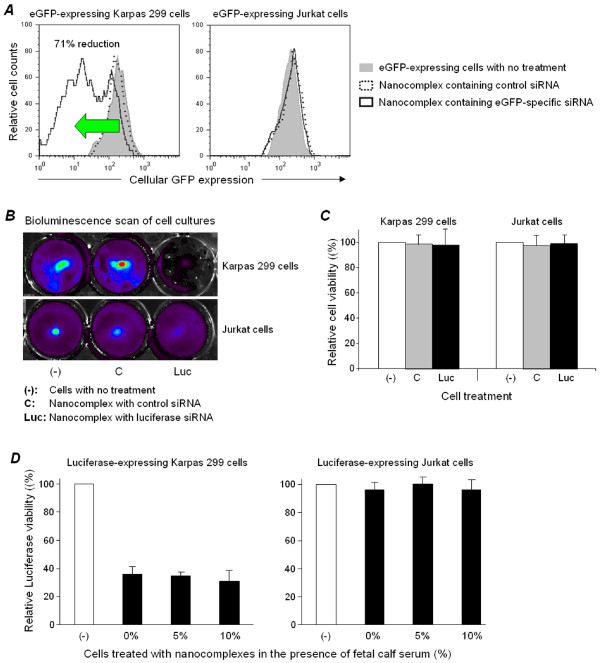
**Lymphoma cell type-dependent gene silencing by the nanocomplexes**. **A**, Stably-expressing eGFP and luciferase Karpas 299 and Jurkat cells were used as reporters for the gene silencing studies. The cells were treated with the nanocomplexes containing eGFP siRNA along with the CD30 aptamer, non-relevant control siRNA along with CD30 aptamer, or left untreated for 2 days. Reduction of eGFP expression (%) was quantified by flow cytometry. **B**, Similarly, the cells were treated with nanocomplexes containing luciferase siRNA along with the CD30 aptamer for 2 days. After addition of luciferin into the cultures, the cellular luciferase activity was detected by bioluminescence scanning. **C**, To rule out non-specific cytotoxicity, relative viabilities (%) in the same sets of cells described in **B **were simultaneously examined by counting viable cell numbers. **D**, Cells were treated with the nanocomplexes as described in **B **and also in the presence of 5% or 10% fetal calf serum. After culture for 2 days at 37°C, cellular luciferase activity was detected by bioluminescence scanning.

First, to determine whether siRNAs remain functional after incorporation into the nanocomplexes, we first tested a nanocomplex containing enhanced green fluorescent protein (eGFP)-targeted siRNAs. For quantitative measurement of gene silencing, Karpas 299 and Jurkat reporter cells that stably express eGFP and luciferase gene were utilized [43]. The cells were treated with nanocomplexes containing the CD30 aptamer and eGFP siRNA for 2 days, and changes in eGFP expression were quantified by flow cytometry. As shown in Figure 6A, a 71% reduction in eGFP expression was detected in Karpas 299 cells treated with nanocomplexes containing eGFP-targeted siRNA, but there was no reduction in cells treated with nanocomplexes containing an irrelevant control siRNA. Further, siRNA delivery was CD30 specific, because no change in eGFP expression was observed in CD30 negative Jurkat cells after eGFP siRNA-containing nanocomplex treatment. We also demonstrated that gene silencing was not limited to eGFP by making nanocomplexes containing siRNA specific for the luciferase gene and CD30 aptamer for ALCL targeting. Cells were treated with the luciferase-specific siRNA nanocomplexes and 2 days post-treatment, luciferin was added to the cell cultures and luciferase activity detected by bioluminescence scanning. Treatment with the nanocomplex selectively silenced the luciferase gene in Karpas 299 cells (a 76% reduction in cellular luciferase activity), but not in CD30-negative Jurkat cells (Figure 6B). To rule out non-specific cytotoxicity of the nanocomplexes, cell viability was simultaneously monitored. As before, exposure of Karpas 299 cells and Jurkat cells to the nanocomplexes had no effect on cell viability (Figure 6C). Moreover, cells were treated with the nanocomplexes for luciferase gene silencing as described above and also in the presence of fetal calf serum. Gene silencing studies showed that the presence of 10% serum had no effect on the nanocomplex-induced lymphoma cell type-dependent gene silencing (Figure 6D), further confirming the biostability of the formulated nanocomplexes.

### Nanocomplex treatment silences ALK expression and causes growth arrest of ALCL cells

**Figure 7:**
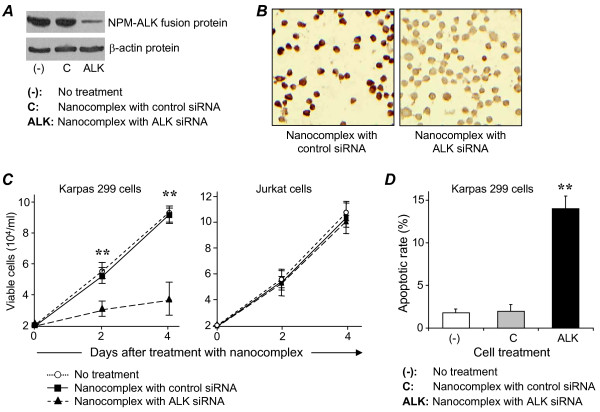
**ALK gene-silencing and growth inhibition of ALCL cells by functional RNA nanocomplexes**. **A**, Cultured Karpas 299 cells were treated with the nanocomplex containing both ALK siRNA and CD30 aptamer for 4 days. Cellular proteins were then separated by electrophoresis and ALK fusion proteins (NPM-ALK) were detected by immunoblotting. Cellular β-actin protein expression was also measured as an internal control for gene expression. **B**, Cellular ALK fusion protein expression in the same set of treated Karpas 299 cells was also simultaneously detected by immunocytochemical staining. **C**, To study the corresponding effects on cellular proliferation and viability when the ALK gene was silenced, Karpas 299 and control Jurkat cells were treated with the nanocomplexes containing ALK siRNA or irrelevant control siRNA, or were not treated. The number of viable cells was counted under each treatment condition on days 2 and 4 post-treatment. **D**, To assess apoptosis, Karpas 299 cells were treated as described above for 2 days and then stained with FITC-conjugated Annexin V. The number of apoptotic cells (%) was measured by flow cytometry. ** *P *< 0.05.

To determine if nanocomplex-mediated delivery of an ALK-targeted siRNA could knockdown gene expression, we assembled nanocomplexes by incorporating both CD30 aptamer and ALK siRNA into the PEI-citrate carrier (Figure 1A). Cultured Karpas 299 cells were treated with the nanocomplex for 2 days and ALK gene silencing was monitored by immunoblotting with an anti-ALK protein antibody. Treating cells with nanocomplexes containing ALK siRNA resulted in specific knockdown of the nucelophosmin-ALK (NPM-ALK) fusion protein expression but did not affect cellular β-actin expression, used as an internal control (Figure 7A). Additionally, immunocytochemical staining of nanocomplex-treated Karpas 299 cells was performed to assess NPM-ALK fusion protein expression (Figure 7B). As with the immunoblotting analysis, a marked decrease in NPM-ALK protein expression was observed in cells treated with the ALK-siRNA nanocomplexes but not with nanocomplexes containing am irrelevant control siRNA.

To examine changes in Karpas 299 cell viability, cells were treated with ALK-siRNA nanocomplexes, as described above, and growth kinetics and cell viability were simultaneously measured at 2 and 4 days post-treatment. Cells were treated for 4 days because the cellular NPM-ALK fusion protein has a long half life time, ≥48 hours. As shown in Figure 7C, treating Karpas 299 cells with the nanocomplex significantly inhibited cell growth (*P *< 0.05). In contrast, the growth kinetics of nanocomplex-treated Jurkat cells was unaffected. To assess apoptosis, Karpas 299 cells were treated with the nanocomplex for 24 hours, as described above, stained with FITC-conjugated Annexin V, and analyzed by flow cytometry. Nanocomplex-treatment significantly increased the percentage of apoptotic cells from a basal level of 2.2% to 14.1% (Figure 7D, *P *< 0.05).

## Discussion

An ideal *in vivo *siRNA carrier system will safely transport the 'cargo' to the desired destination, release a functional cargo in a tissue/cell specific manner, and have no off-target or adverse drug effects. In this study, we have developed this type of carrier system by formulating a functional RNA nanocomplex that is both tumor cell type-selective and cancer gene-specific for ALCL. Advantages of these nanocomplexes include: 1) incorporating siRNAs into a nano-sized carrier will increase their physical size and could prevent the rapid elimination of siRNA from the blood circulation *in vivo*; 2) incorporating CD30 aptamers will enable specific accumulation of the nanocomplexes within tumor sites and eliminate potential off target side effects of the nanocomplex components; and 3) it is possible to incorporate more than one siRNA and/or therapeutic drug into the nanocomplex to generate additive or synergistic repressive effects on tumor cells. The use of specific ligands for cell targeting and reduction of the PEI dose is critical for *in vivo *feasibility. PEI polymers have been used for cell transfection at concentrations ranging from 5 to 10 μg/ml [15-19], at which moderate cytotoxicity has been reported [20,21]. As demonstrated in this study, incorporating the CD30 aptamer allowed us to use a sub-toxic dose of the PEI carrier in the nanocomplex, less than 1/20 of the reported cytotoxic concentration. It is notable that under *in vivo *conditions, the CD30 aptamer-mediated cell binding will likely result in an accumulation of the nanocomplexes exclusively in lymphoma tumor tissues and increase the local PEI concentration, possibly reaching a toxic dose. Interestingly, the increased PEI concentration within tumor tissues may enhance the *in vivo *therapeutic effect of the nanocomplex, but have no adverse effect on normal tissues.

## Conclusions

In this study, we have described an approach for developing therapeutic agent by formulating a nanocomplex that is both tumor cell-selective and cancer gene-specific for ALCL. The nanocomplexes are specific and non-cytotoxic to lymphoma cells, which advance great potential for their clinical applications.

## Methods

### Chemical reagents and oligonucleotide synthesis

Branched polyethyleneimine (60 kDa) was purchased from Sigma-Aldrich (Catalog #P3143, St. Louis, MO). Sodium citrate was obtained from Fisher Scientific (Pittsburgh, PA). For silencing the enhanced green fluorescent protein gene (eGFP), eGFP-targeted siRNA was purchased along with a paired control siRNA from Ambion (catalog # AM4626, Foster City, CA). The ALK-targeted siRNA was synthesized by Ambion using the reported sequences: sense, 5'-CACUUAGUAGUGUACCGCCtt-3' and antisense, 5'-GGCGGUACACUACUAAGUGtt-3' [38]. A reporter for the cell binding assays was constructed by synthesizing a single-stranded DNA (ssDNA) oligonucleotide containing the sense ALK siRNA sequence conjugated at the 5' end to the fluorochrome Cy5 (excitation 645 nm/emission 665). The CD30 aptamer was synthesized by Bio-Synthesis (Lewisville, TX), as previously described [31] using the following sequence: 5'-GAUUCGUAUGGGUGGGAUCGG GAAGGGCUACGAACACCG-3'.

### Formulation and characterization of the nanocomplex

To generate the PEI polymer carrier, we used sodium citrate to crosslink PEI molecules. The PEI-citrate core structure (nanocore) was formed by mixing one part by volume of a 100 μg/ml pH 6.0 PEI solution with six parts by volume of sodium citrate. To obtain PEI-citrate nanocores of the optimal size, different 'R' ratios (defined as the ratio of the number of carboxylate groups from citrate to the number of primary amine groups from PEI) were tested. These ratios ranged from 10 to 1 and were obtained by changing the concentration of the citrate solution. The size of the PEI-citrate nanocores produced for each R ratio was determined by obtaining dynamic light scattering measurement (DLS) using a Brookhaven ZetaPALS with a BI-9000AT digital autocorrelator at a wavelength of 656 nm. Diameters were obtained by fitting DLS correlation with the CONTIN routine available through the instrument software 9KDLSW. Electrophoretic mobility was also determined with ZetaPALS using a dip-in (Uzgiris type) electrode in 4-mL polystyrene cuvettes, and the zeta potential was calculated using the Smoluchowski model. To assemble the nanocomplex, three parts by volume of synthetic CD30 aptamers (10nM) and siRNAs (100nM) (or Cy5-labeled ssDNA for validation purposes) were added to the nanocore reaction 5 minutes after initiation and were incorporated into the PEI-citrate nanocores through non-covalent charge forces (Figure 1A). To confirm the colloidal stability of the assembled nanocomplexes, they were incubated in RPMI 1640 cell culture medium at room temperature and the nanocomplex size was monitored by DLS over time.

### Cell binding assays

Karpas 299 cells (a human CD30-expressing ALCL cell line from the German Collection of Microorganisms and Cell Cultures (DSMZ, Braunschweig) and Jurkat cells (a CD30-negative human leukemia/lymphoma cell line from ATCC, Manassas, VA) were used in this study. Cells (2 × 10^5^) were incubated with PEI-citrate, PEI-citrate/ssDNA(Cy5), or PEI-citrate/ssDNA(Cy5)/Aptamer, as indicated in Figure 5, in 0.5 ml of culture medium for 30 minutes at room temperature. Cell binding of the nanocomplexes was analyzed by flow cytometry (LSRII, BD Biosciences) and fluorescent microscopy (Olympus IX71 inverted microscope) to detect cell surface Cy5 signal. To test their biostability, nanocomplexes were incubated in RPMI 1640 medium, and CD30 aptamer-mediated cell binding was examined at the indicated intervals over 24 hours.

### *In vitro *functional assays

Cytotoxicity assay: the individual components were added into Karpas 299 cell cultures (2 × 10^5^/sample) at their maximal concentrations: 100nM CD30 aptamer, 100 nM ALK gene-targeting siRNA, and 4.2 μM sodium citrate (pH 6.0). After 48 hours, cells were harvested, and cytotoxicity was evaluated by flow cytometry using forward and side scatter parameters. PEI toxicity was also examined by adding serial dilution ranging from 5.48 μg/ml to 0.027 μg/ml to the cell cultures and evaluated as described above. Cell viability assay: cells (2 × 10^5^/sample) were treated as indicated and stained with 0.1% trypan blue in PBS for 15 minutes. Viable cells were counted using a hemocytometer and light microscope. The relative rate of cell growth was determined by calculating the ratio of the number of viable cells in treated samples to the number of cells in the control samples (no treatment). Cell apoptosis assay: cells (2 × 10^5^/sample) were treated for 24 hours as indicated and stained with FITC-conjugated Annexin V using a kit from BD Biosciences. Apoptotic cells were detected by flow cytometry.

### Confocal fluorescence microscopy

To demonstrate cell-selective intracellular delivery of the nanocomplex, cultured cells (2 × 10^5^/sample) were treated with the nanocomplex diluted in 0.5 ml of RPMI 1640 medium without serum for 4 hours at 37°C. Cells were then washed twice with PBS and stained with 1 μg/ml 4'-6-diamidino-2-phenylindole (DAPI, Invitrogen) for 15 minutes to label nuclei. Lastly, cells were cytospined onto slide and examined using a laser scanning confocal microscope (Olympus, Fluo ViewTM 1000) at 400 × magnification.

### Gene silencing studies

To validate ALCL-selective gene silencing, an eGFP-specific nanocomplex was generated as indicated in Figure 1B and incubated with 2 × 10^5 ^eGFP-expressing Karpas 299 or Jurkat cells (40) at PEI concentration of 0.274 μg/ml in 0.5 ml of RPMI 1640 medium for 4 hours at 37°C with or without fetal calf serum as indicated in the figures. The cells were then washed twice with PBS and cultured in medium containing 10% FBS. Expression of eGFP was evaluated by flow cytometry on day 2 post-treatment. Nanocomplexes containing luciferase-targeted siRNAs (Ambion, Austin, TX) were also tested in the luciferase-transfected Karpas 299 or Jurkat cells (40). Changes in luciferase activity in the cultures were evaluated by bioluminescence scanning.

To silence ALK expression, cultured Karpas 299 cells were treated with nanocomplexes containing ALK-targeted siRNAs at a PEI concentration of 0.274 μg/ml, as described above. eGFP-specific nanocomplexes were utilized as an irrelevant gene silencing control. For immunocytochemical studies, cells were harvested at day 2 post-treatment, and cytospins were prepared with a cell preparation kit (BD Biosciences). The cellular nucelophosmin-ALK fusion protein was detected using a mouse anti-human ALK antibody (1:300 dilution, BD Biosciences) and visualized with the DAKO ChemMate detection kit using a horseradish peroxidase-conjugated rabbit anti-mouse antibody and the color development substrate DAB. Images were taken using a light microscope. In addition, ALK fusion protein expression in the treated cells was examined by immunoblotting, as previously described [43].

### Statistical analysis

All experiments were performed greater than or equal to three times. Data were analyzed by Student's *t *test. *P *values of less than 0.05 were considered significant.

## Competing interests

The authors declare that they have no competing interests.

## Authors' contributions

NZ conducted majority of experiments and participated in the design of the study. HGB and MSW conceived the PEI-citrate nanocore concept. Additionally, HGB optimized the PEI-citrate nanocore size and performed the physical characterization of the nanocomplex. MSW. participated in the experimental design and review of the manuscript. YZ designed the experiments and wrote the final manuscript. All authors read and approved the final manuscript.

## Supplementary Material

Additional file 1 **Electron microscopy of the nanocomplexes**. Approximately 2 μl of the nanocomplex solution composed of PEI-citrate nanocores, ALK siRNA, and the CD30 aptamer were dried on an ultrathin carbon film on a carbon support with holes and imaged with a JEOL 1230 high contrast transmission electron microscope operating at an accelerating voltage of 120 V. The arrow points to a nanocomplex. 200 Click here for file
