# Supplementary material for: A nanocomplex that is both tumor cell-selective and cancer gene-specific for anaplastic large cell lymphoma
Source: J Nanobiotechnology. 2011 Jan 31;9:2. doi: 10.1186/1477-3155-9-2 (PMC3045295; doi:10.1186/1477-3155-9-2)

Additional files

Additional file 1

Title: Electron microscopy of the nanocomplexes.

Approximately 2 µl of the nanocomplex solution composed of PEI-citrate nanocores, ALK siRNA, and the CD30 aptamer were dried on an ultrathin carbon film on a carbon support with holes and imaged with a JEOL 1230 high contrast transmission electron microscope operating at an accelerating voltage of 120 V. The arrow points to a nanocomplex.

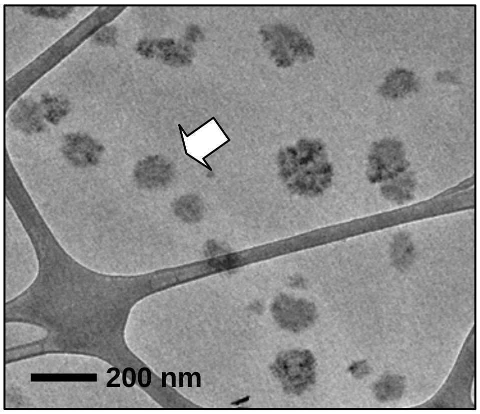

Supplement: Additional file 1 — Electron microscopy of the nanocomplexes. Approximately 2 μl of the nanocomplex solution composed of PEI-citrate nanocores, ALK siRNA, and the CD30 aptamer were dried on an ultrathin carbon film on a carbon support with holes and imaged with a JEOL 1230 high contrast transmission electron microscope operating at an accelerating voltage of 120 V. The arrow points to a nanocomplex. 200 [file 1477-3155-9-2-S1.PDF]
